# Tinnitus Phenotypes and Their Response to the Enriched Acoustic Environment (EAE) Treatment

**DOI:** 10.3390/brainsci16080894

**Published:** 2026-08-21

**Authors:** Charles Bouzou, Marta Fernández-Ledesma, María Cuesta, Pedro Cobo

**Affiliations:** 1 Ecole Centrale Mediterranée, 38 Rue Frédéric Joliot-Curie, 13013 Marseille, France; charles.bouzou@centrale-med.fr; 2 Faculty of Biomedical and Health Sciences, Universidad Europea de Madrid, Campus de Villaviciosa, Calle Tajo s/n, Villaviciosa de Odón, 28670 Madrid, Spain; marta.fernandez2@universidadeuropea.es; 3 Institute of Physical and Information Technologies (ITEFI), CSIC, Serrano 144, 28006 Madrid, Spain; m.cuesta@csic.es

**Keywords:** tinnitus severity, tinnitus treatment, tinnitus phenotypes

## Abstract

**Highlights:**

**What are the main findings?**

- Five tinnitus phenotypes were obtained by applying a data-driven K-clustering technique, based on demographic (age, gender), hearing (average auditory threshold, hearing loss pattern), tinnitus (duration, severity, lateralization, type of sound, aetiology) and emotional (anxiety/depression) variables, to a retrospective cross-sectional cohort of 564 tinnitus subjects.
- The analysis of the response of these five phenotypes to an Enriched Acoustic Environment (EAE) treatment revealed significant differences in the composition of the excluded, dropout, and non-improved groups. However, minor differences were found within the improved group.

**What are the implications of the main findings?**

- This study confirmed the marked heterogeneity of tinnitus patients regarding hearing health, tinnitus characteristics, emotional symptoms, and tinnitus-related distress.
- The identified phenotypes differed not only in hearing loss patterns and tinnitus characteristics, but also in emotional burden and tinnitus severity.

**Abstract:**

**Background/Objectives**: The aim of this study was to apply data-driven clustering techniques for the subtyping of tinnitus severity to a retrospective cross-sectional cohort of 564 subjects. Additionally, the response of the resulting phenotypes to an enriched acoustic environment (EAE) treatment was investigated. **Methods**: A hierarchical decision cascade was applied to classify the measured audiograms of participants into six hearing loss (HL) patterns. K-clustering techniques were applied to demographic (gender), hearing (average audiometric threshold, HL pattern), tinnitus (tinnitus onset age, duration, severity, lateralisation, type of sound, aetiology) and emotional variables. Subjects received customised EAE stimulus to be listened to for one hour daily over four months. **Results**: Five phenotypes were found by applying K-clustering techniques to numerical and categorical variables. EAE provided an average tinnitus severity reduction of 24.3 points, which was clinically relevant and statistically significant, in 78% of subjects who completed the four-month treatment. **Conclusions**: Analysis of the performance of EAE treatment in the different phenotypes afforded clear differences in the composition of excluded, dropped out, and not improved subjects. Absolute mean improvement of both severity and emotional scores was larger in phenotypes PH3 and PH4, as they had higher tinnitus distress and emotional symptoms at baseline. Nevertheless, relative change of severity score was notably smaller in PH4.

## 1. Introduction

Tinnitus is the perception of a sound that has not been generated by any source, external or internal to the body [1]. In many patients, it arises as the consequence of plastic mechanisms in the neural auditory pathway to compensate for deafferentation from the peripheral auditory system [2]. It is an auditory symptom affecting roughly 10–15% of the population that becomes a disorder when it produces severe emotional burden in the subject [3]. Approximately 1–2% of people suffer from this disorder, producing severe emotional troubles such as stress, anxiety, and/or depression [4]. In spite of this high prevalence, nowadays there is no cure, neither pharmacological nor of other type, for tinnitus [5].

This neural sound is highly heterogeneous, and can be perceived as a ringing, hissing or tonal sound, located towards the left, right, or both ears, with low or high pitch [6]. Cederroth et al. [7] proposed four dimensions of tinnitus heterogeneity, related to its perception (laterality, pitch, sound type, etc.), multiple risk factors (hearing loss (HL), neck and temporomandibular junction (TMJ) disorders, aging, etc.) and comorbidities (hyperacusis, depression, headache, distress), which can differ largely among patients, and the response to treatments.

**Figure 1:**
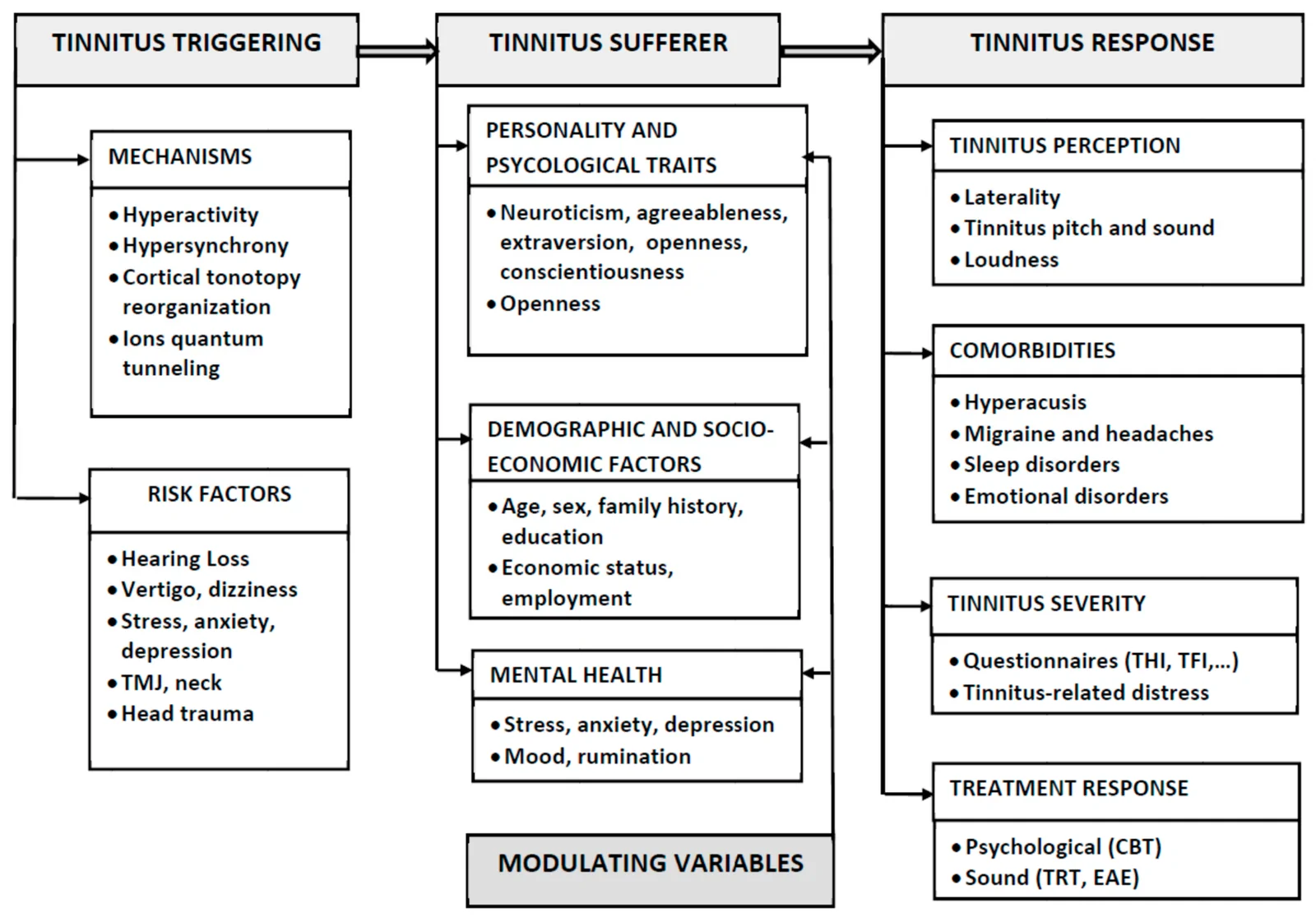
Input–output model of tinnitus heterogeneity.

Tinnitus heterogeneity may also be explained by means of an input–output model (IOM) (see Figure 1). Regarding the triggering of tinnitus, several possible mechanisms have been proposed, including cortical tonotopic map reorganisation, hypersynchrony, hyperactivity [2], and ion quantum tunnelling [8], and tinnitus risk factors [9], comprising hearing loss, vertigo and dizziness, emotional disorders (stress, anxiety, depression), neck and temporomandibular joint (TMJ) disorders, or head traumas.

The tinnitus sufferer is also highly heterogeneous, as he/she is modulated by several variables [10], such as personality and psychological traits (neuroticism, agreeableness, extraversion, openness, conscientiousness) [11], demographic and socio-economic factors (age, sex, family history, education, economic status, employment) [10], and mental health (stress, anxiety, depression, mood, rumination) [12].

The response to tinnitus is likewise rather heterogeneous regarding its perception (pitch and loudness, laterality), comorbidities (hyperacusis, sleep and emotional disorders, migraine and headache) [13], severity (tinnitus-related distress, as measured by tinnitus questionnaires) [14,15], and response to treatments such as cognitive behavioural therapy (CBT) [16], tinnitus retraining therapy (TRT) [17], and enriched acoustic environment (EAE) [18].

As a consequence of this heterogeneity, different subtypes (or phenotypes) of tinnitus may exist, each one with distinct clinical profiles [7]. Therefore, disentangling these subtypes may be important to enhance our understanding of tinnitus as well as for improving the diagnosis and the design of personalised treatments [19,20], as tinnitus heterogeneity could be one of the causes of their low overall success [21].

According to Genitsaridi et al. [10], there are three analysis categories to perform tinnitus subtyping: hypothesis-driven, where subgrouping is based on the criterion of the researcher, and the characteristics of the resulting groups are investigated subsequently, data-driven, where multidimensional statistical analysis is applied to the available data of participants to find distinct clusters or classes [22], and treatment response-driven, where participants are allocated to different subgroups depending on their response to some specific treatment [23].

On the other hand, although there is currently no cure for tinnitus, many treatments have been proposed to reduce tinnitus-related distress. The most used treatments are cognitive behavioural therapy (CBT) [16] and tinnitus retraining therapy (TRT) [17]. TRT is based on the neurophysiological model of Jastreboff and combines counselling with sound therapy [17]. According to this model, tinnitus has two components: the emotional one, consequence of the exacerbation of the network between the neural part of the auditory system and the limbic system, and the auditory one, due to the hyperactivity on the auditory path from the periphery to the auditory cortex. While counselling is aimed at reducing the exacerbation of the limbic-auditory network, this in turn decreasing the emotional symptoms of tinnitus, the sound therapy aims to reduce the strength of the neural activity related to tinnitus.

The TRT uses broadband noise as sound therapy [17]. Other alternative sounds have been proposed, including filtered broadband noise combined with music, narrowband noise, matched or not to the tinnitus pitch, tones at frequencies excluding the tinnitus pitch, tones matched to the tinnitus pitch, etc. [24]. One of these alternative treatments, named enriched acoustic environment (EAE), has been recently proposed and combines counselling with a sound therapy consisting of sequential (tone-pip, tone-burst, or tone-gamma) or continuous stimuli matched to the hearing loss (HL) of the subject, that must be heard one hour per day for four consecutive months [25,26,27] Compared to TRT, EAE provide similar results (23% of tinnitus distress release in the 83% of people completing the treatment) but with 90% less sound exposure time [28].

Although the above scores are mean values, individual results are rather variables. Some of the subjects quit the treatment before completing the four-month period, others do not respond to the treatment (they do not reduce their tinnitus-related distress), and many others reduce their distress in rather inconsistent amounts. This variable response to treatments among subjects can be attributed to the tinnitus heterogeneity [23,29,30].

The aim of this work was twofold. On the one hand, data-driven tinnitus phenotyping was carried out, based on the application of K-clustering techniques to hearing (averaged auditory threshold (*AAT*) and predominant HL pattern), tinnitus (lateralisation, sound type, predominant aetiology, tinnitus onset age, tinnitus duration, tinnitus severity), emotional (anxiety, depression), and demographic (gender, age) data of 564 tinnitus subjects. Concerning the hearing data, while hearing loss is a clear risk factor for triggering tinnitus, a very weak correlation has been shown to exist between the *AAT*, a measure of hearing loss, and the Tinnitus Handicap Inventory (*THI*), a measure of tinnitus distress [6]. Therefore, the hearing condition should not be introduced in the K-clustering algorithm as a numerical variable. It can also be introduced as a categorical variable, closely related to the HL curve (the audiogram), specifically as a HL pattern. On the other hand, the response of these phenotypes to EAE treatment was analysed.

## 2. Materials and Methods

### 2.1. Participants

A total of 564 subjects were recruited for a clinical study on sound therapies of tinnitus in two centres. A total 425 of them were engaged through the study at the ITEFI-CSIC and 139 came from the study at the European University of Madrid. All participants signed an Informed Consent form. Only participants fulfilling selection criteria were considered for the study. Those younger than 18 and older than 80 years, with any recent surgical operation in the ears, with recent Ménière episode, with tinnitus-related distress, as measured by the Tinnitus Handicap Inventory (*THI*) (see Section 2.3), lower than 20, and with depressive symptoms score, as measured by the Hospital Anxiety and Depression Scale (*HADS-A*) (see Section 2.4), larger than 18, were excluded. After applying these selection criteria, 80 subjects (14.3%) were excluded.

### 2.2. Hearing Levels and Hearing Patterns

Hearing levels (HLs) of participants were measured in an audiometric room at 11 frequencies from 125 Hz to 8 kHz. The resulting audiograms were automatically categorised using a custom-written classification algorithm in RStudio 2026.06.1. The program has three phases:

First, the algorithm divided the acoustic spectrum into three frequency bands:

- Low Frequencies (LFs): 125 to 500 Hz
- Mid Frequencies (MFs): 750 to 2000 Hz
- High Frequencies (HFs): 3000 to 8000 Hz

Average audiometric thresholds (*AAT*) were calculated for each frequency band, as well as for the whole frequency interval, as

(1) $$AAT=\frac{1}{11}\sum_{125\;Hz}^{8\;kHz}{HL}_{i}\left(f\right)$$

(2) $${AAT}_{LF}=\frac{1}{3}\sum_{125\;Hz}^{500\;Hz}{HL}_{i}\left(f\right)$$

(3) $${AAT}_{MF}=\frac{1}{4}\sum_{750\;Hz}^{2\;kHz}{HL}_{i}\left(f\right)$$

(4) $${AAT}_{HF}=\frac{1}{4}\sum_{3\;kHz}^{8\;kHz}{HL}_{i}\left(f\right)$$

which served as the basis for pattern calculations.

Secondly, for each ear, the program identified a specific audiometric profile by applying a hierarchical decision cascade:

- Priority 1 (Localised Dips): Detection of a sharp threshold drop relative to its adjacent frequencies. The curve was classified as a scotoma (a single notch, typically around 4000 Hz) or sawtooth (multiple notches).
- Priority 2 (U-shaped Profiles): Detection of curvature. A concave profile was assigned if mid-frequencies were better preserved than the extremes; conversely, a convex profile was assigned if mid-frequencies were the most severely affected.
- Priority 3 (Slopes): In the absence of a U-shaped configuration, the algorithm evaluated whether the hearing loss was predominantly localised within either the LF or HF ranges.
- Priority 4 (Flat or Normal Profiles): For flat configurations, the program decided between flat profile (homogeneous overall hearing loss) and a normal profile (hearing loss under 20 dB across all frequencies).
- Priority 5 (Complex Profiles): If none of the mentioned criteria were met, the audiogram was classified as complex.

Finally, the algorithm compared the results from both ears to give a global diagnosis, following the next rules:

- Asymmetric: If the mean threshold difference between the two ears exceeded 15 dB, the final diagnosis was automatically categorised as asymmetric.
- Unification by Similarity: If both ears showed high point-by-point correlation (an interaural difference of less than 6 dB at each frequency) but one ear was classified as complex, the algorithm assigned the profile of the other ear to both.
- Clinical Dominance: In the presence of different profiles for the two ears without significant level asymmetry (less than 15 dB), the final diagnosis was the profile ranked highest in the step 2 hierarchy.

### 2.3. Tinnitus Characteristics

Tinnitus duration, tinnitus lateralisation (bilateral, left ear, right ear), possible aetiology and comorbidities (hyperacusis, vertigo, temporomandibular junction (TMJ) or neck disorders) were obtained directly from the participants through a Tinnitus Assessment Form. This form was filled out and anticipated by the participants after recruitment and revised and completed during a direct interview before treatment. The tinnitus onset age (*TOA* = Age − Tinnitus Duration) was calculated from age and tinnitus duration.

Tinnitus severity was assessed through two questionnaires, the Tinnitus Handicap Inventory (*THI*) [14,15], and the Tinnitus Functional Index (*TFI*) [31,32]. Spanish versions of *THI* [33] and *TFI* (provided by the Oregon Health & Science University) were utilised. According to the *THI* score, tinnitus was graded as slight (*THI* 0–16), mild (*THI* 18–36), moderate (*THI* 38–56), severe (*THI* 58–76) or catastrophic (*THI* 78–100) [15]. Both questionnaires are patient-reported outcome measures (PROM) and show a high overlap [34].

The type of tinnitus sound and the tinnitus pitch was were measured using a self-developed application [18]. The tinnitus subject was exposed to a band-pass filtered noise. The centre of the filter established the tinnitus frequency. The width of the band-pass defined the sound type. Bandwidths of 0.1%, lower than 9% and higher than 10% of the frequency centre defined tonal, ringing or hissing sounds, respectively. The type of sound was obtained by matching the own tinnitus of the subject to the best band-filtered noise generated by this application.

### 2.4. Emotional Symptoms

Tinnitus-related distress is highly correlated with emotional symptoms such as stress, anxiety and depression [35,36,37]. Anxiety and depression symptoms have been assessed through the Hospital Anxiety and Depression Scale (*HADS*) [38,39]. The *HADS* consists of 14 questions, 7 concerning anxiety (*HADS-A*) and 7 concerning depression (*HADS-D*), with 4 possible responses scored as 0, 1, 2, or 3. Thus, the *HADS-A* and *HADS-D* range from 0 to 21. *HADS-A* and *HADS-D* were rated as normal (scores 0–7), mild (scores 8–10), moderate (scores 11–15), and severe (scores 16–21).

### 2.5. Tinnitus Phenotypes

Tinnitus subtyping was carried out by the use of K-prototype, a clustering algorithm [22] by R. K-prototype clustering constitutes a hybrid unsupervised learning solution specifically designed to process heterogeneous databases combining numerical and categorical variables, unlike the more classic K-means, which only processes numerical variables. It works by defining a unified cost function that combines two metrics: the squared Euclidean distance for numerical variables and a dissimilarity measure (based on the Hamming coefficient) for categorical variables.

Unlike a simple selection of existing points, the algorithm proceeded by iterative optimisation. After an initialization initialisation where N individuals were randomly chosen as starting points, the algorithm refined these prototypes at each iteration: it calculated the mean of the continuous attributes and the mode (the most frequent value) of the discrete attributes for each formed group. This iterative process continues until convergence, minimising global inertia (maximising the internal cohesion of the groups).

This procedure was repeated multiple times, each time with different starting patients (the N participants chosen randomly). Ultimately, the algorithm only retained the trial with the lowest inertia. This approach allowed for robust segmentation, and was capable of identifying clinical profiles whose characteristics were both numerical and categorical. It should be noted, however, that the algorithm does not choose the number of clusters in the sample itself; it was therefore necessary to determine this number beforehand.

The other tool used in tinnitus subtyping was the principal component analysis (PCA). This consists of a statistical dimensionality reduction method aimed at transforming a set of initial variables, which may be correlated, into a system of new orthogonal variables called principal components (PC). Mathematically, this process relies on a linear transformation that projects the data into a new space where the first component (PC1) captures the maximum variance of the dataset, while the subsequent components (PC2, PC3, etc.) explain decreasing portions of the residual variance under the constraint of orthogonality.

The main advantage of PCA in the analysis of complex data is twofold: on one hand, it allows for the synthesis of information by reducing statistical noise, and, on the other, it enables the visualization visualisation of the underlying structure of the data in a space containing a reduced number of dimensions. By retaining only those components whose eigenvalues—that is, the portions of variance they explain—are above a certain threshold, the variability of the sample can thus be modelled in a simplified manner without significant loss of information, and the sample can be visualised in the principal component space, thereby facilitating the interpretation of clusters.

### 2.6. EAE Treatment

EAE treatment is based on the neurophysiological model of Jastreboff [17] and combines counselling with sound therapy. Counselling was applied in a unique session at the beginning of the treatment and consisted of an explanation to the subject of the functioning of the auditory system, as well as the mechanisms, epidemiology, aetiology and possible treatments of tinnitus. Sound therapy was applied over four months and involved the hearing of a customised EAE sound stimulus for one hour every day. The EAE sound stimulus can be sequential or continuous. Sequential EAE sounds are sequences of tone-pip [40], tone-burst [41] or tone-gamma [25,26] with random frequency within the hearing frequency band (125 Hz–8 kHz) and amplitude proportional to the HL value at this frequency. Continuous EAE are broadband sounds matched to the hearing loss curves [42]. The subject was asked to choose one of these four customised sounds based on their comfort. The chosen sound was given to the participant in mp3 format, with the recommendation of listening to it with headphones one hour per day for four months.

## 3. Results

### 3.1. Demographic Data

The number of subjects, mean and standard deviation (SD) of all participants, including their classification by sex, are summarised in Table 1. Men in this cohort were more frequent (61.5%) and were slightly older (52.9 years) than women.

### 3.2. Hearing Levels and Hearing Patterns

**Figure 2:**
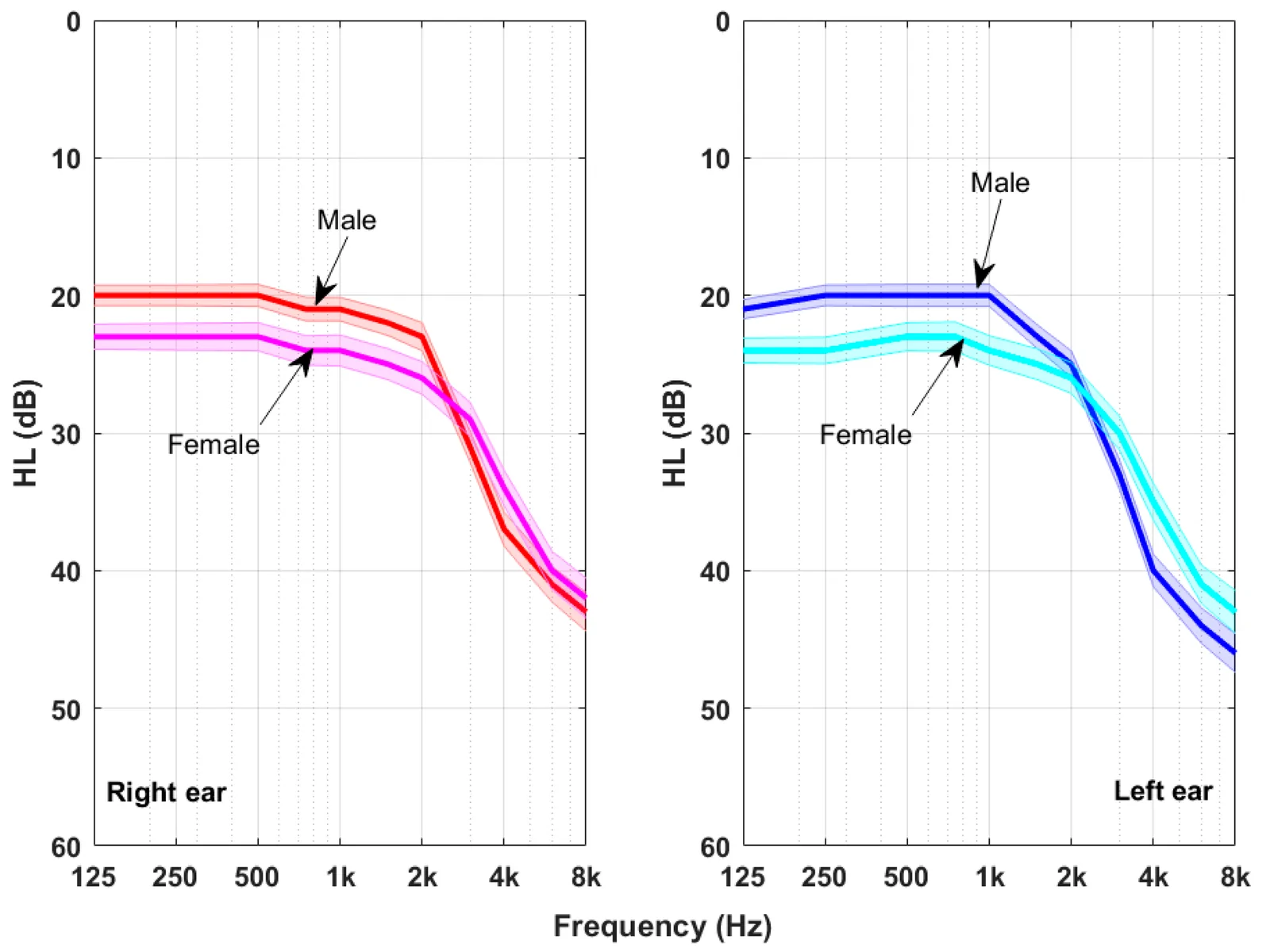
Mean HL curves of participants by sex. Shaded area around the HL curves outlines confidence intervals.

Figure 2 shows the average HL curves of the male and female participants. On average, both exhibited high-frequency hearing losses, higher in the left ear. Furthermore, women had higher mean hearing losses than men below 3000 Hz, but lower mean hearing losses above this frequency.

The algorithm described in Section 2.2 detected six HL patterns: normal hearing (NH), high frequency (HF) losses, scotoma (SCT), asymmetric (ASM) HL, concave/convex (CVX), and complex (CPX) HL (see Table 2).

A total of 50 out of 564 (8.9%) had normal hearing, 154/564 (27.3%) displayed high frequency HL, 145/564 (25.7%) exhibited a scotoma in some ear, 96/564 (17.0%) showed an asymmetric hearing, 66/564 (11.7%) had concave or convex HL patterns, and the HL of the remaining 53/564 (9.4%) were included in the complex subgroup.

### 3.3. Tinnitus Characteristics and Emotional Symptoms

Tinnitus and emotional data from the participants were collected as numerical (*TOA*, tinnitus duration, tinnitus severity (*THI*, *TFI*), emotional burden (*HADS-A*, *HADS-D*), tinnitus frequency) and categorical (tinnitus sound type, tinnitus lateralisation, aetiology) variables.

Tinnitus frequency was not found to affect tinnitus phenotypes and will not be used in the following. 55.8% of participants had bilateral tinnitus (55.8% males, 56.0% females), 27.0% perceived their tinnitus in the left ear (26.3% males, 28% females), and 17.2% located their tinnitus in the right ear (17.9% males, 16.0% females). Concerning sex differences, whilst lateralisation was almost identical for men and women, tonal tinnitus was much more frequent in males (56.9%) than in females (42.2%).

Table 3 summarises the mean and SD values of the numerical variables. On average, participants debuted with their tinnitus with 44.2 years (43.6 males, 45.2 females) [43], which lasted 8.2 years (9.4 males, 6.4 females), had moderate (*THI* = 50.1, *TFI* = 52.0) severity (*THI* = 49.5 and *TFI* = 50.1 males, *THI* = 51.2 and *TFI* = 55.0 females), and emotional burden of *HADS-A* = 8.5 and *HADS-D* = 6.1 (*HADS-A* = 8.1 and *HADS-D* = 6.1 for males, *HADS-A* = 9.1 and *HADS-D* = 6.2 for females).

**Figure 3:**
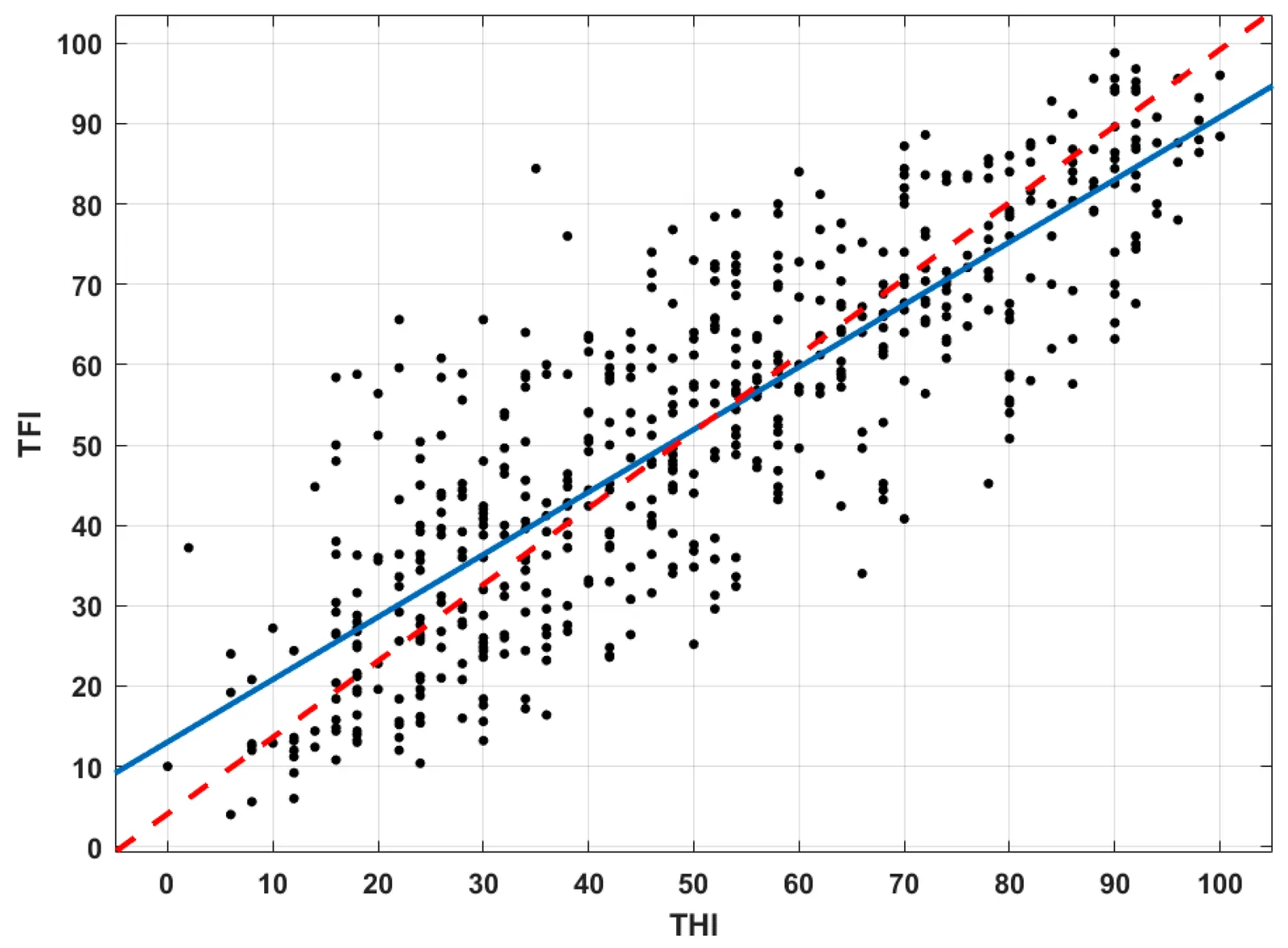
Scatter plot of *THI* versus *TFI* scores.

Since *THI* and *TFI* measure tinnitus severity, their scores should be similar [44]. While both questionnaires were designed and validated to assess tinnitus severity at baseline, *TFI* was furthermore optimised for responsiveness to changes in outcomes related to intervention [32]. But, at baseline, both questionnaire scores provide very similar scores, as shown in Figure 3. The fitted (solid blue) and the diagonal (*THI* = *TFI*, dashed red) lines are shown for comparison. Both scores were highly correlated (r = 0.84) around the diagonal, although *THI* scores were slightly higher for *THI* < 50 and slightly lower for *THI* > 50. Although most values are close to the diagonal, some others, which can be considered outlier scores, are far from this line. These outliers can be moved towards the diagonal by defining a new numerical variable *THFI* = (*THI* + *TFI*)/2, which will be used in subsequent analyses.

The variables of tinnitus severity (*THI* and *TFI*) and emotional symptoms (*HADS-A* and *HADS-D*) are known to be highly correlated [45]. Table 4 shows the correlation coefficients of these variables for the 564 participants in this cohort. The variables *HADS* = *HADS-A* + *HADS-D* and *THFI* = (*THI* + *TFI*)/2 are also included. The strongest correlation was obtained between the *THFI* and the *HADS*. Therefore, these variables were chosen to account for tinnitus severity and emotional symptoms.

### 3.4. Tinnitus Phenotypes

The K-clustering algorithm grouped the 564 patients into clusters representing the different tinnitus phenotypes. Five phenotypes were obtained by applying K-clustering techniques to demographic (*TOA*, gender), hearing (*AAT*, HL pattern), tinnitus (tinnitus duration, *THFI*, lateralisation, type of sound, aetiology) and emotional (*HADS*) variables. The most relevant number of clusters was determined using the elbow method, which evaluated the evolution of the cost function (the sum of distances within the groups) as a function of the number of clusters, K, and choosing the K for which adding an extra group no longer improved notably the internal consistency. This elbow marked the optimal balance between statistical precision and the simplicity of interpreting the results. There was a trade-off between using too many phenotypes, which may lead to overfitting, and too few phenotypes, which may result in information loss and increased heterogeneity among groups.

To ensure an equitable contribution of numerical (*TOA*, Duration, *AAT*, *THFI*, *HADS*) and categorical (gender, lateralisation, sound type, aetiology, HL pattern) variables, the weighting parameter, *λ*, was used via the *lambdaest* function of R. The final weight applied was *λ* = 1.447404. This calibration step was essential to prevent numerical variables with high variance from overshadowing the influence of qualitative criteria during segmentation. This technique afforded K = 5 phenotypes.

To evaluate the quality of the produced segmentation, a hybrid cohesion score was calculated for each cluster by adding the dispersion of numerical variables and the entropy of categorical variables. Numerical variables in the program were normalised (standard deviation divided by the mean). For categorical variables, the function measured Shannon’s entropy, which quantifies the degree of label purity (here HL pattern, aetiology, localisation, sound type, and gender) within the group, with zero entropy indicating that all individuals shared identical attributes.

To visually verify the existence of these five phenotypes and refine their characteristics, a principal component analysis (PCA) was conducted using the MEDA Toolbox in MATLAB R2023a [46]. PCA was used to ensure that the identified groups were not only homogeneous but also sufficiently distinct from one another to justify their existence. This analysis involved finding principal components (PCs)—linear combinations of real numerical variables—that maximised the total variance of the sample. In this study, these PC were linear combinations of age, mean *THFI* score, tinnitus duration, *AAT*, and *HADS* score. The PCA constructed these components independently of the individual phenotype assignments. Individuals were then color-coded according to their phenotypes on a 3D projection. Variables were auto-scaled (centred and reduced) to ensure they had equal weight.

**Figure 4:**
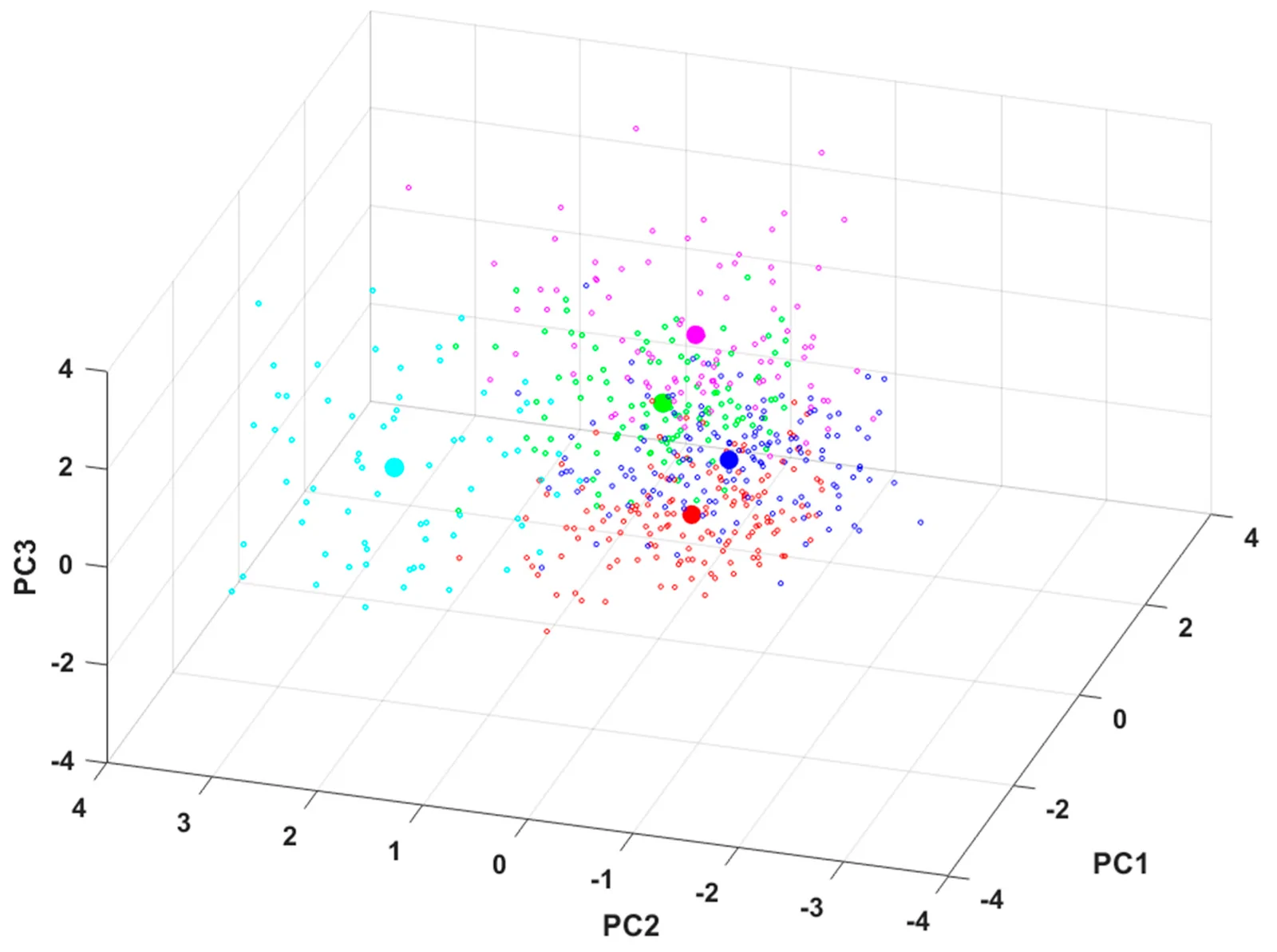
Scatter plot of used variables in the Principal Components coordinate system for phenotypes PH1 (blue), PH2 (red), PH3 (green), PH4 (magenta) and PH5 (cyan). Centroids (bigger filled dots) are also shown for comparison.

Figure 4 shows the distribution of participants across the first three principal components, according to their assigned phenotype. Centroids of each phenotype are also shown as bigger filled dots for comparison. Separation between centroids is a measure of how distinct these five clusters truly are from one another. Although partial overlap was observed, it can be seen that the phenotypes tended to occupy different regions of the reduced-dimensional space, suggesting that the identified clusters represent groups with distinct clinical characteristics.

Some outliers can be observed in Figure 4, which were categorised into two types: Q and D. Category D represents patients with extreme but consistent data within the sample (e.g., a patient with very high THFI and HADS scores). Category Q represents patients who contradict the logic of the sample (e.g., a patient with a very high THFI but a very low HADS score). The small number of Q outliers indicated a clear population dynamic, facilitating segmentation. The use of PCA thus confirmed that the clusters were separable and graphically coherent, according to the visualisation of the distribution of the sample in a 3D space.

Finally, the five phenotypes summarised in Table 5 were obtained. The cohesion score of each phenotype was included in the last row of Table 5. According to these scores, the clusters were equally coherent, thus validating the relevance of the chosen number of groups (K = 5).

PH1 was the most frequent phenotype in this cohort (26.4%), including subjects with mild high-frequency losses, medium tinnitus duration (7.3 years), mild tinnitus distress, normal anxiety/depression score, with predominant male gender, bilateral tonal sound, and HL aetiology. PH2 (25.2%) was composed of subjects with more premature tinnitus onset, lower tinnitus duration, lower average auditory threshold, moderate tinnitus distress, and slightly higher *HADS* score. These subjects were also predominantly male, with bilateral tonal sound, scotoma as the HL pattern, and emotional troubles as the most expected aetiology. The more relevant features of patients in the PH3 phenotype (19.3%) were the high tinnitus-related distress and emotional scores. Thus, the predominant aetiology in this subgroup was emotional troubles. The PH4 phenotype (16.5%) was characterised by subjects with predominant asymmetric HL. They perceived mainly a hissing sound in the left ear. They also had severe tinnitus distress and high HADS scores. Another notable difference with the other subgroups was the predominantly female gender of these subjects. Finally, subjects of the PH5 phenotype (12.6%) had remarkably lower tinnitus onset age and longer tinnitus duration. They also had moderate tinnitus distress, normal anxiety/depression scores, predominant scotoma, and bilateral tonal sound.

### 3.5. Results of EAE Therapy

**Figure 5:**
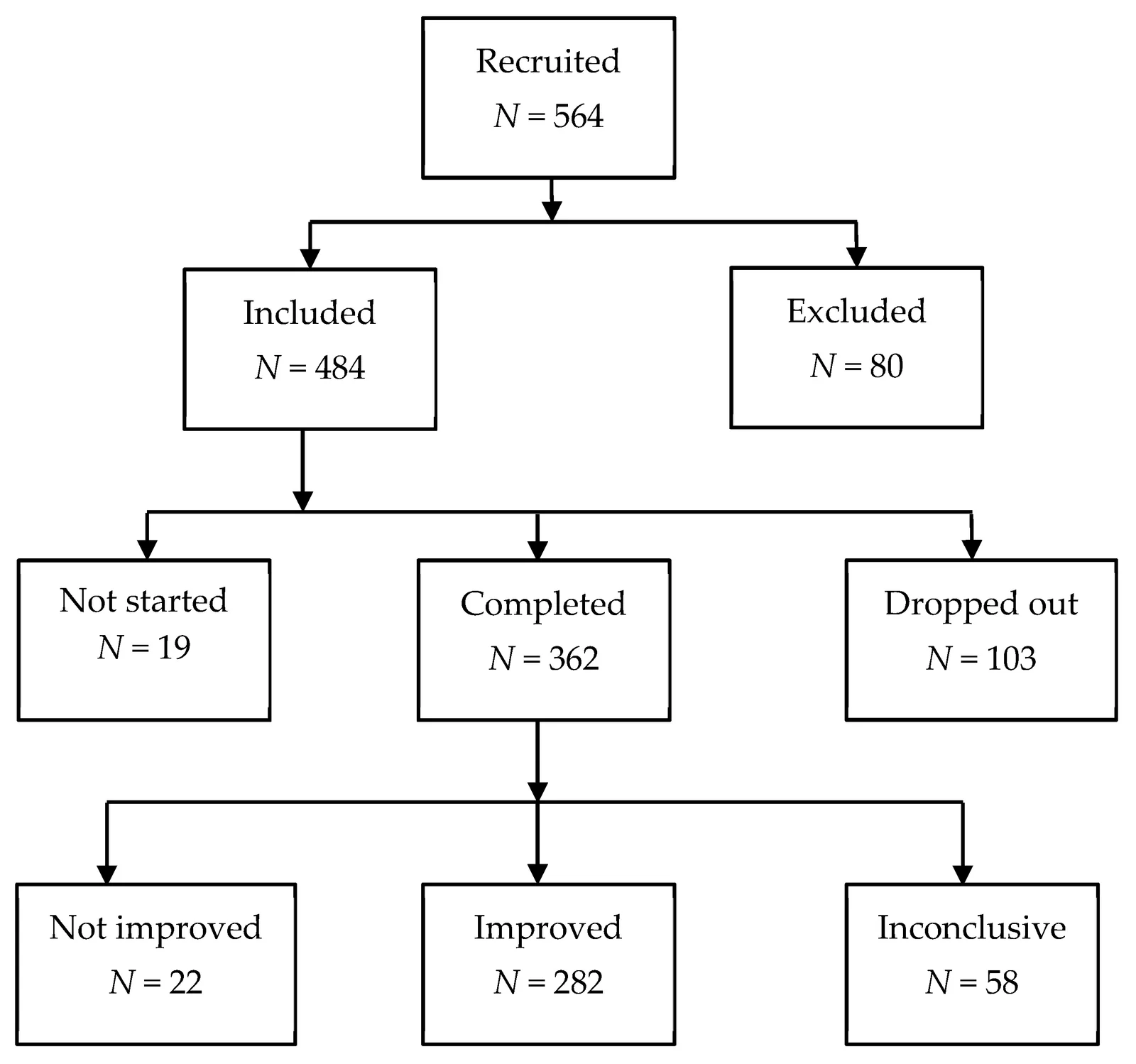
Flow diagram of participants in the EAE study.

Figure 5 shows the flow diagram of participants in the EAE study. Of the 564 recruited participants, 80 (14.2%) were excluded based on the eligibility criteria. Of the 484 included participants, 19 (3.4% of the total recruited sample) attended the counselling session but decided not to initiate EAE sound therapy, 103 (18.3%) dropped out before completing the four-month treatment, and 362 (64.2%) completed the EAE treatment. Of these, 22 (6%) did not improve (neither *THI* nor *TFI* were reduced), 58 (16%) were inconclusive (one of *THI* or *TFI* decreased but the other increased), and 282 (78%) improved (both the *THI* and *TFI* diminished).

Table 6 summarises the number and percentages of subjects excluded, not started, dropped out, not improved, inconclusive and improved in each phenotype. Almost half (48.8%) of excluded subjects belonged to phenotype PH1. The largest percentage (31.6%) of not started people were of phenotype PH4. 28.1% of participants who dropped out of the treatment were of phenotype PH3. Not improved subjects were mainly (40.9%) from phenotype PH1. 31% of inconclusive people fitted also in phenotype PH1. Percentages of improved subjects did not differ much from those in the cohort.

**Figure 6:**
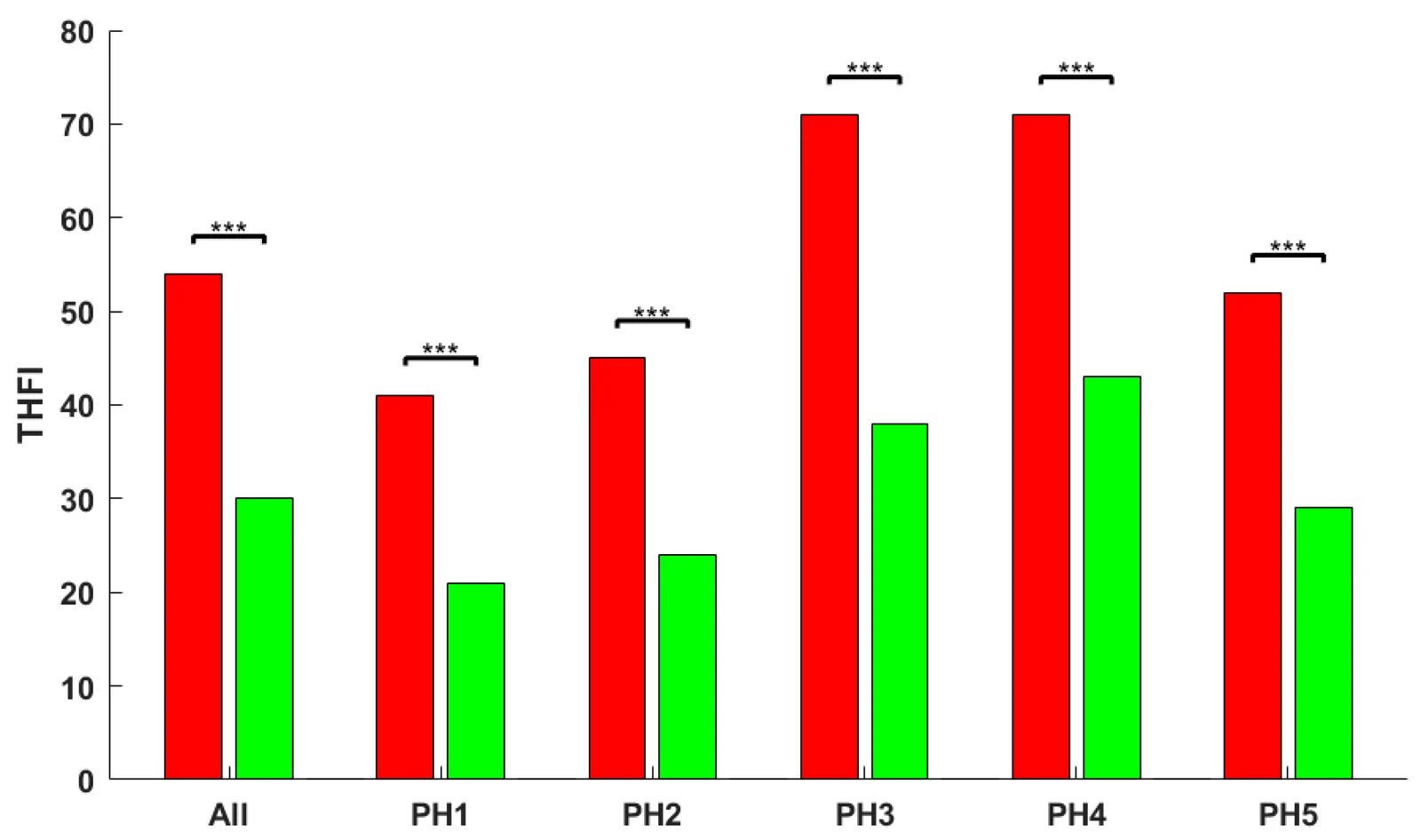
Bar plot of mean THFI values before (red bars) and after (green bars) EAE treatment for each phenotype. Three stars (***) means *p* < 0.001.

**Figure 7:**
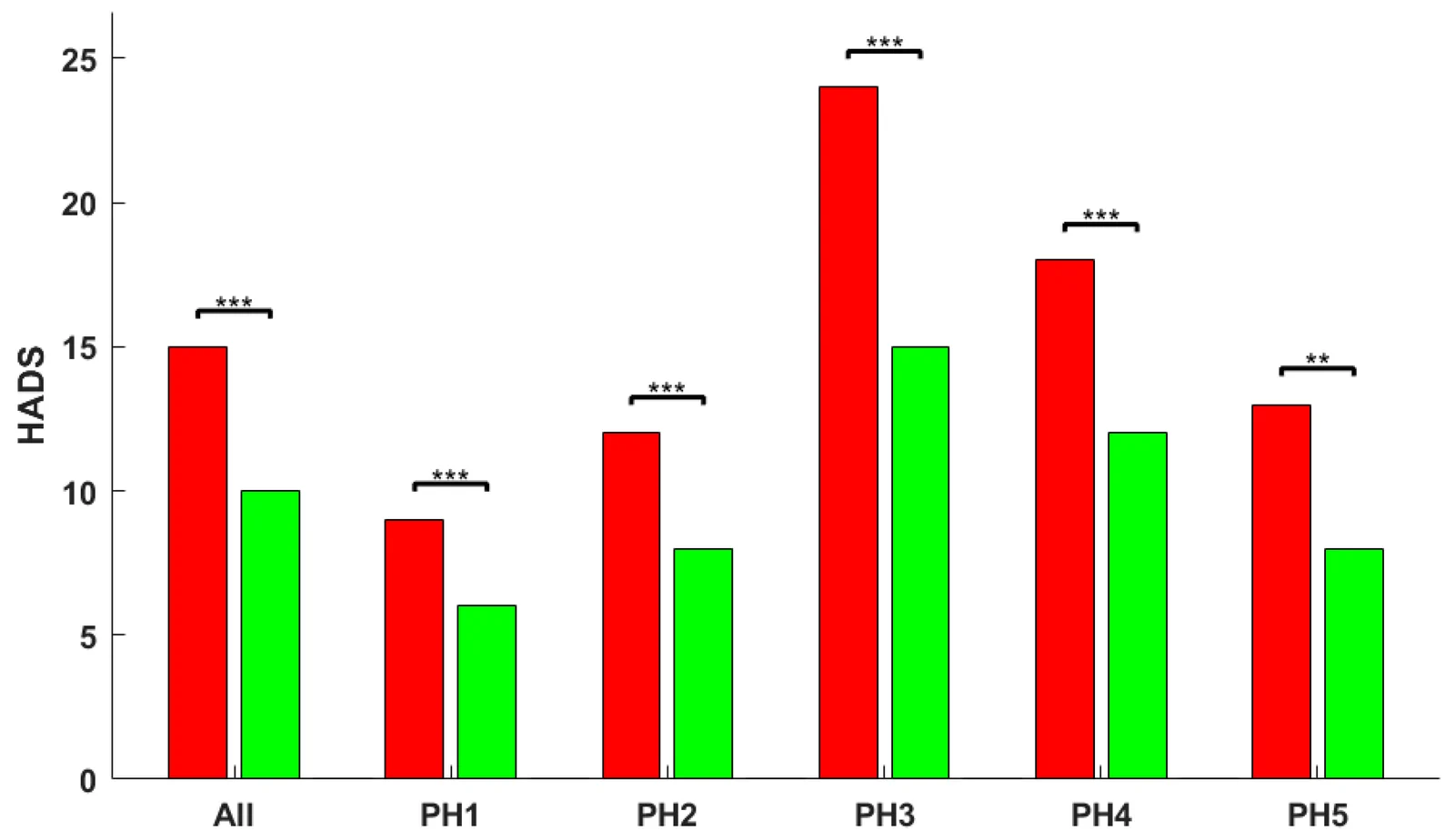
Bar plot of mean HDS values before (red bars) and after (green bars) EAE treatment for each phenotype. Three stars (***) means *p* < 0.001 and two stars (**) means *p* < 0.01.

Figure 6 and Figure 7 show the bar plots of THFI and HADS scores, before and after the EAE treatment, for all participants and for each phenotype. Paired *t*-tests, without correction for multiple comparisons, were applied to tinnitus-related distress and emotional symptoms reductions assessed with *THFI* and *HADS*, respectively. The null hypothesis was that both stimuli provide equal mean reduction at the 5% significance level. Both scales provide statistically different mean improvement after EAE treatment for all phenotypes.

The null hypothesis was that both stimuli provide equal mean reduction at the 5% significance level. Both scales provide statistically different mean improvement after EAE treatment for all phenotypes. Stars above bars denote significant differences between scores before and after EAE treatment. Three stars (***) means *p* < 0.001. Therefore, reductions in both *THFI* and *HADS* scores following EAE treatment were statistically significant (*p* < 0.001).

Table 7 summarises the mean, SD, Cohen’s d, and confidence interval values of Δ*THFI* = *THFI*_ini_ − *THFI*_fin_ and ΔHADS = *HADS_i_*_ni_ − *HADS*_fin_ scores for all participants and for each phenotype. An average tinnitus related-distress decrease of 24.4 points was achieved, which is clinically relevant and statistically significant [18,45]. Absolute and relative changes in *THFI* and *HADS* scores varied across phenotypes. PH3 and PH4 showed the largest absolute *THFI* reductions, whereas the highest relative *THFI* reduction (mean decrease of the score/mean baseline value of the score) was observed in PH1 and the lowest in PH4. Despite these differences, all phenotypes demonstrated clinically relevant improvements following EAE treatment. Concerning emotional symptoms (*HADS*), PH3 showed both the largest absolute and relative reduction. Significant pre-post reductions were observed in all phenotypes.

## 4. Discussion

The demographic profile of this clinical tinnitus cohort aligned with established epidemiological patterns showing increased tinnitus prevalence with age and a male predominance in clinical samples [47,48]. The mean age of 52.4 years places this sample in middle-to-late adulthood, consistent with epidemiological evidence showing a progressive increase in tinnitus prevalence with age [47]. Notably, the mean *TOA* was approximately 44 years in both sexes, suggesting that tinnitus typically emerges in mid-life. This *TOA* was slightly younger than the mean sample age, indicating an average tinnitus duration of about 8 years, in line with the chronic nature of tinnitus in treatment-seeking populations [49].

Some sex-related differences were also observed in this cohort. Males reported slightly higher mean tinnitus pitch and longer tinnitus duration, whereas females showed higher *THI*, *TFI*, and anxiety scores. This pattern is consistent with previous studies showing higher prevalence of tinnitus in men, commonly attributed to greater noise exposure, but greater tinnitus-related distress and psychological burden among women in clinical populations [11,28,36,48]. In contrast, depression scores did not vary significantly by sex in the present sample, differing from previous reports of higher depressive symptom levels among women with tinnitus [50].

The predominance of bilateral (55.8%) and tonal (51.3%) tinnitus in this cohort matched the phenotypic profile described in large descriptive series and phenotyping studies. Clinical cohorts report that tinnitus is most often perceived as bilateral or “in-the-head”, with tonal percept representing the most common sound type [51,52].

The subjects of this cohort had mean high-frequency hearing losses, suggesting age-related and noise-related hearing losses. However, when the HL curves were more closely analysed to look for characteristic HL patterns, other HL shapes emerged. Although losses related to aging (27%) and noise overexposure (26%) were still the majority, asymmetric HL (17%), usually associated with Ménière disease and sudden HL, concave/convex (12%), often with genetic origin, and normal HL (9%) were found. Langguth et al. [53] classified the HL of 2838 patients in four grades: normal hearing, mild/moderate hearing, severe/profound hearing, and no data available. In a similar way, Kassjański et al. [54] automatically classified the HL of 15,046 patients in four subtypes: normal hearing, conductive HL, sensorineural HL, and mixed HL. Therefore, their categorisation tried to reflect the grade/type of hearing rather than looking for different HL patterns.

The association between tinnitus severity (*THI*/*TFI*) and symptoms of anxiety and depression (*HADS-A*/*HADS-D*) observed in our sample is consistent with previous evidence. Multiple studies have shown that tinnitus-related distress is closely linked to emotional comorbidity. Cross-sectional and longitudinal studies across both clinical and population-based cohorts have reported higher levels of anxiety, depression, somatisation, and reduced quality of life among individuals with tinnitus, particularly in those reporting greater tinnitus-related distress [28,55,56].

Higher *THI*/*TFI* scores were associated with elevated anxiety and depressive symptoms. This association is consistent with psychological models describing tinnitus distress as a vicious cycle involving negative appraisal, selective attention, physiological arousal, and mood dysregulation [4,57,58,59,60]. In line with this framework, recent evidence suggested that emotional distress can interfere with central auditory processing and speech-in-noise performance in individuals with tinnitus, indicating that anxiety-driven attentional capture by the phantom percept may divert cognitive resources from complex listening tasks [61].

We are using *THI* and *TFI* questionnaires to assess tinnitus-related distress. While both questionnaires have been designed and validated to assess tinnitus severity at baseline [14,15,31,32], *TFI* was furthermore optimised for “responsiveness to changes in outcomes related to intervention” [32]. But, at baseline, both questionnaires should provide very similar scores, as shown in Figure 3. Using *THFI* as the numerical variable for the K-clustering algorithm moves towards the bisector of the (*THI*, *TFI*) plane (the dashed red line in Figure 3), the “outlier scores”. Therefore, it is important to emphasise that we are using *THFI* as an exploratory measure rather than a validated clinical instrument.

Our K-clustering technique, based on numerical and categorical variables (demographic (age, gender), hearing health (*AAT*, HL pattern), tinnitus severity (*THFI*), emotional symptoms (*HADS*), and tinnitus characteristics (duration, lateralisation, sound type, aetiology)), provided five characteristic phenotypes. As it can be seen in Table 5, two phenotypes (PH1 and PH3) have high frequency HL pattern (as 27.3% (154/564) of participants), two phenotypes (PH2 and PH5) are characterised by a scotoma (as 25.7% (145/164) of participants), and one phenotype (PH4) has asymmetric HL (as 17% (96/564) of participants). Therefore, the HL pattern has a clear influence on the obtained phenotypes. Our five phenotypes differed from the four phenotypes found by Niemman et al. [30] (avoidant, psychosomatic, somatic and distress subgroups), as they were based on a different set of questionnaires for assessing tinnitus distress, emotional troubles, psychosomatic features and personality traits obtained from 1228 subjects. Kim et al. [62] applied K-means clustering to other variables (pure tone average (PTA), uncomfortable loudness levels, *THI*, visual analog scales for subjective symptoms, Beck Depression Inventory (*BDI*), and Beck Anxiety Inventory) measured in 311 patients, obtaining three tinnitus subtypes (severe tinnitus with normal hearing, hearing loss dominant tinnitus, and mild psychosomatic tinnitus). As expected, tinnitus subtypes seem to depend on the variables assessed from the participants.

Our analysis of EAE outcomes across the different phenotypes revealed differences in the composition of the excluded, dropout, and non-improved groups. Almost half (48.8%) of the excluded and 40.9% of the not improved people belonged to the PH1 phenotype. Among the participants who dropped out, 28.1% belonged to the PH3 phenotype. However, the distribution of improved participants across the five phenotypes was broadly similar to the phenotype distribution observed in the overall cohort. Absolute mean improvement of both *THFI* and *HADS* was larger in phenotypes PH3 and PH4, as they had higher tinnitus distress and emotional symptoms at baseline [18,44]. Nevertheless, relative change in *THFI* score was notably smaller in PH4. Despite the differences in the magnitude of absolute and relative changes, reductions in *THFI* and *HADS* scores were observed across all phenotypes. This result suggests that EAE treatment may lead to improvements across different tinnitus profiles rather than being limited to a specific phenotype.

The phenotypes also showed that similar levels of tinnitus-related distress may occur in participants with different clinical characteristics. For instance, PH3 and PH4 had similar baseline *THFI* scores but differed in gender distribution, hearing status, tinnitus lateralisation, sound type, and predominant aetiology. These findings support the multidimensional nature of tinnitus and suggest that severity scores alone may not fully characterise the clinical profile of participants. Therefore, data-driven phenotyping may provide complementary information for the clinical characterisation of tinnitus beyond that obtained from severity questionnaires alone.

Several limitations should be considered when interpreting these findings. First, the absence of a control group limits causal inference regarding the effects of EAE. Without a control group to account for placebo effects or natural habituation over the four-month period, the average reduction in *THFI* score should be considered of observational nature rather than causal. This issue will be addressed in future research. Second, treatment outcomes were evaluated among participants who completed the four-month intervention, and treatment discontinuation may have introduced selection bias. Third, the identified phenotypes were derived and evaluated within the same cohort and therefore require validation in independent populations. As with any data-driven clustering approach, the resulting phenotypes may depend on the variables included and the methodological decisions applied. Finally, some clinical variables, including the presumed tinnitus aetiology, were based on information reported by the participants.

Future studies should externally validate the identified phenotypes and assess their stability across independent clinical populations. These analyses may help determine whether data-driven tinnitus phenotyping can contribute to treatment selection and the development of more personalised therapeutic approaches.

## 5. Conclusions

This study confirmed the marked heterogeneity of tinnitus patients regarding hearing health, tinnitus characteristics, emotional symptoms, and tinnitus-related distress. By combining demographic, audiological, perceptual, and emotional variables through a data-driven K-prototype clustering approach, five differentiated tinnitus phenotypes were identified in a large retrospective cohort of 564 participants.

The identified phenotypes differed not only in hearing loss patterns and tinnitus characteristics, but also in emotional burden and tinnitus severity. While some subgroups were mainly associated with high-frequency hearing loss and mild distress, others were characterised by predominant emotional troubles, severe tinnitus handicap, elevated anxiety/depression scores, or asymmetric hearing losses. Furthermore, sex differences were observed across phenotypes, with female participants exhibiting greater tinnitus severity and emotional symptoms despite similar tinnitus onset ages. These findings support the hypothesis that tinnitus severity cannot be exclusively explained by auditory dysfunction, but rather emerges from the interaction between hearing status, emotional factors, and individual clinical characteristics.

An overall tinnitus severity reduction of 24.3 points, clinically relevant and statistically significant, was found in 78% of participants completing the four-month EAE treatment. Some differences were found in the response to these five phenotypes. The percentages of people in the subgroups that improved after EAE were similar to those of the cohort, although almost half of the subjects excluded and not improved belonged to PH1. Although absolute improvements were better in phenotypes with larger tinnitus severity scores at baseline, relative changes were larger in the PH1 phenotype.

## Figures and Tables

**Table 1:** Age and sex of participants.

	All	Male	Female
*N* (%)	564 (100%)	347 (61.5%)	217 (38.5%)
Mean age (years)	52.4	52.9	51.6
SD age (years)	11.4	10.6	12.4

**Table 2:** Number of patients for the different HL patterns.

HL Pattern	*N*	HL Example
Normal Hearing	50	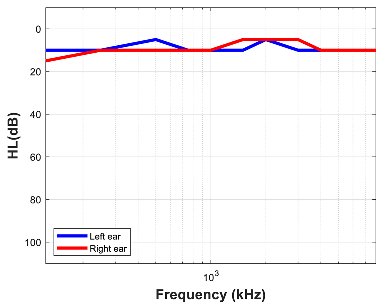
High Frequency Loss	154	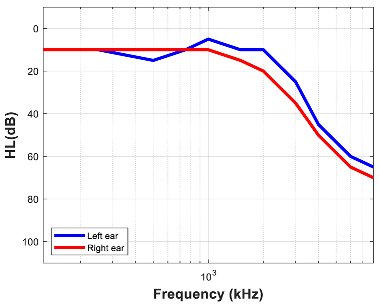
Scotoma	145	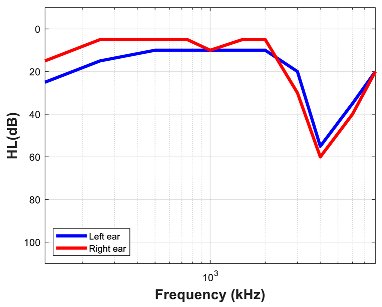
Asymmetric	96	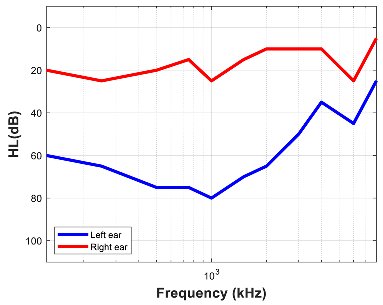
Convex/Concave	66	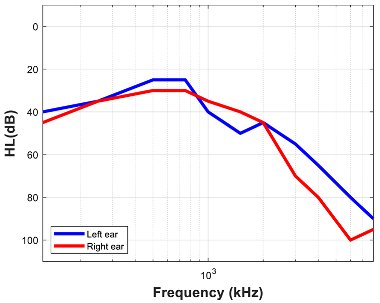
Complex	53	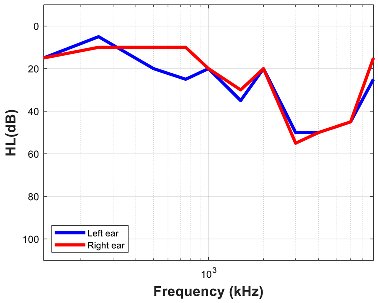

**Table 3:** Mean and SD values of *TOA*, tinnitus duration, *THI*, *TFI*, *HADS-A*, and *HADS-D*.

		*TOA* (Years)	Duration (Years)	*THI*	*TFI*	*HADS-A*	*HADS-D*
All	Mean	44.2	8.2	50.1	52.0	8.5	6.1
	SD	12.8	9.5	23.7	22.0	4.4	4.2
Males	Mean	43.6	9.4	49.5	50.1	8.1	6.1
	SD	12.6	10.2	23.9	21.7	4.3	4.2
Females	Mean	45.2	6.4	51.2	55.0	9.1	6.2
	SD	13.0	7.8	23.5	22.1	4.5	4.2

**Table 4:** Correlation coefficients between tinnitus severity and emotional symptoms.

		Tinnitus Severity		
**Emotional symptoms**		*THI*	*TFI*	*THFI*
	*HADS-A*	0.69	0.63	0.69
	*HADS-D*	0.68	0.63	0.69
	*HADS*	0.73	0.67	0.74

**Table 5:** Tinnitus phenotypes.

	Phenotypes				
	PH1	PH2	PH3	PH4	PH5
*N* (%)	149 (26.4%)	142 (25.2%)	109 (19.3%)	93 (16.5%)	71 (12.6%)
*TOA* (years)	52.5	41.4	42.5	51.3	25.7
Duration (years)	7.3	4.9	4.1	4.8	27.5
*AAT* (dB)	33.5	16.7	20.9	42.3	33.4
*THFI*	35.6	40.3	71.7	71.8	46.4
*HADS*	8.5	11.3	24.1	19.8	12.5
Gender (% male/% female)	68/32	60/40	69/31	34/66	75/25
Predominant lateralisation	Bilateral (58%)	Bilateral (52%)	Bilateral (70%)	Left ear (47%)	Bilateral (72%)
Predominant sound type	Tonal (52%)	Tonal (54%)	Tonal (64%)	Hissing (59%)	Tonal (61%)
Predominant HL pattern	High frequency loss (50%)	Scotoma (42%)	High frequency loss (35%)	Asymmetric (58%)	Scotoma (35%)
Predominant aetiology	Hearing loss (76%)	Emotional troubles (57%)	Emotional troubles (50%)	Hearing loss (72%)	Hearing loss (55%)
Cohesion score	7	8	7.5	7.2	7.2

**Table 6:** Number and percentage of subjects in each phenotype.

Phenotype (*N*/Percentage)	Excluded *N* = 80	Not Started *N* = 19	Dropped Out *N* = 103	Not Improved *N* = 22	Inconclusive *N* = 58	Improved *N* = 282
PH1 (149/26.5%)	39 48.8%	2 15.8%	11 10.7%	9 40.9%	18 31.0%	70 24.8%
PH2 (142/25.2%)	18 22.6%	3 10.5%	28 27.2%	3 13.6%	10 17.2%	80 28.4%
PH3 (109/19.0%)	7 8.6%	5 26.3%	29 28.1%	3 13.6%	11 19.0%	54 19.1%
PH4 (93/16.6%)	4 5.0%	6 31.6%	17 16.5%	4 18.2%	10 17.2%	51 18.1%
PH5 (71/12.7%)	12 15.0%	3 15.8%	18 17.5%	3 13.6%	9 15.5%	27 9.6%

**Table 7:** Δ*THFI* and Δ*HADS* score reductions for all participants and for each phenotype.

		All	PH1	PH2	PH3	PH4	PH5
Δ*THFI*	Mean	24.4	20.0	20.8	32.8	28.1	23.0
	SD	14.7	11.0	10.8	16.4	18.8	13.1
	Cohen’s d	1.34	1.74	1.64	2.24	1.49	1.29
	Confidence interval	[1.14, 1.55]	[1.38, 2.10]	[1.26, 2.00]	[1.77, 2.79]	[0.9, 1.96]	[0.69, 1.81]
	Relative change	44.9%	48.6%	46.2%	46.3%	39.4%	44.2%
Δ*HADS*	Mean	5.2	3.1	3.8	9.0	6.3	4.8
	SD	6.0	3.8	4.6	8.0	6.2	5.7
	Cohen’s d	0.76	0.74	0.82	1.35	1.04	0.77
	Confidence interval	[0.60, 0.93]	[0.38, 1.08]	[0.50, 1.14]	[0.90, 1.80]	[0.61, 1.46]	[0.23, 1.27]
	Relative change	34.9%	33.6%	32.4%	38.0%	34.5%	36.5%

## Data Availability

Data are not available for privacy/ethical reasons.
